# Hybrid endoscopic and robotic-assisted resection of a midesophageal schwannoma

**DOI:** 10.1016/j.xjtc.2026.102418

**Published:** 2026-05-14

**Authors:** Colin Rousset, Tomer Lagziel, Roshan Kollipara, Shahriyour Andaz, Stavros N. Stavropoulas, Joshua Melamed

**Affiliations:** a Division of Thoracic Surgery, Icahn School of Medicine at Mount Sinai, Mount Sinai South Nassau, Oceanside, NY; b Division of Gastroenterology, Icahn School of Medicine at Mount Sinai, Mount Sinai South Nassau, Oceanside, NY

**Figure:**
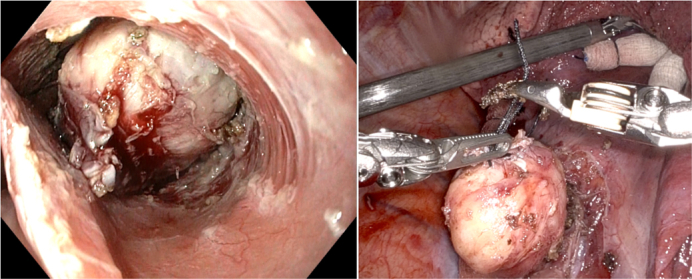
Hybrid endoscopic and robotic resection of midesophageal schwannoma.

**Central Message**

A hybrid endoscopic-robotic approach enables safe, minimally invasive resection of esophageal schwannomas while preserving mucosal integrity and reducing morbidity.

Esophageal schwannomas are rare, benign submucosal tumors originating from Schwann cells of the peripheral nerve sheath.^1^ They most commonly arise in the upper and midesophagus and are frequently discovered incidentally. Because of their submucosal origin and nonspecific imaging features, preoperative diagnosis is often challenging. Resection is indicated for symptomatic or larger lesions. Traditionally managed with thoracotomy or thoracoscopic enucleation,^2^ advances in endoscopic and robotic techniques now allow for minimally invasive, organ-preserving techniques that may reduce morbidity and recovery time.^3^ Here, we report a case of a large midesophageal schwannoma resected using a hybrid endoscopic and robotic-assisted thoracoscopic approach.

## Case Presentation

**Figure 1:**
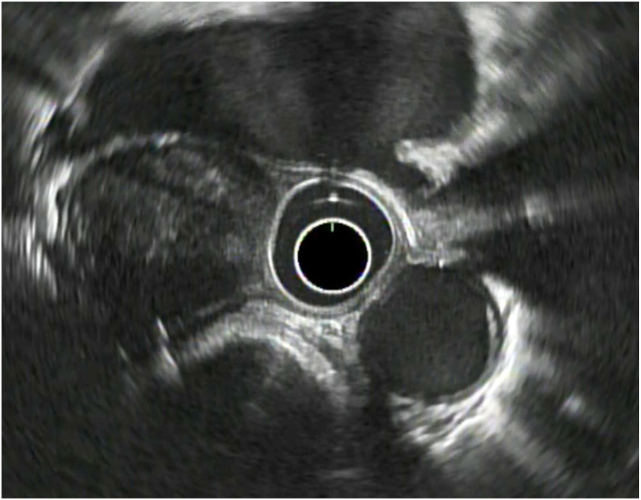
Endoscopic ultrasound of the esophageal lesion. Endoscopic ultrasound demonstrating a well-circumscribed, hypoechoic intramural mass arising from the muscularis propria of the midesophagus, consistent with a subepithelial tumor.

A 69-year-old woman with gastroesophageal reflux disease, hyperlipidemia, and previous tobacco use underwent lung cancer screening computed tomography, which revealed a 4.7- × 2.9-cm intramural midesophageal lesion. She subsequently developed progressive dysphagia, weight loss, and food aversion. Endoscopic ultrasound demonstrated a well-circumscribed hypoechoic mass arising from the muscularis propria without vascular invasion (Figure 1). Fine-needle biopsy revealed spindle cells positive for S100 and negative for desmin and DOG1, consistent with schwannoma.^1^ Given her progressing symptoms and tumor size, resection was recommended. Written informed consent was obtained from the patient for publication of this case report and all associated images and media. Institutional review board approval was not required.

## Surgical Technique

**Figure 2:**
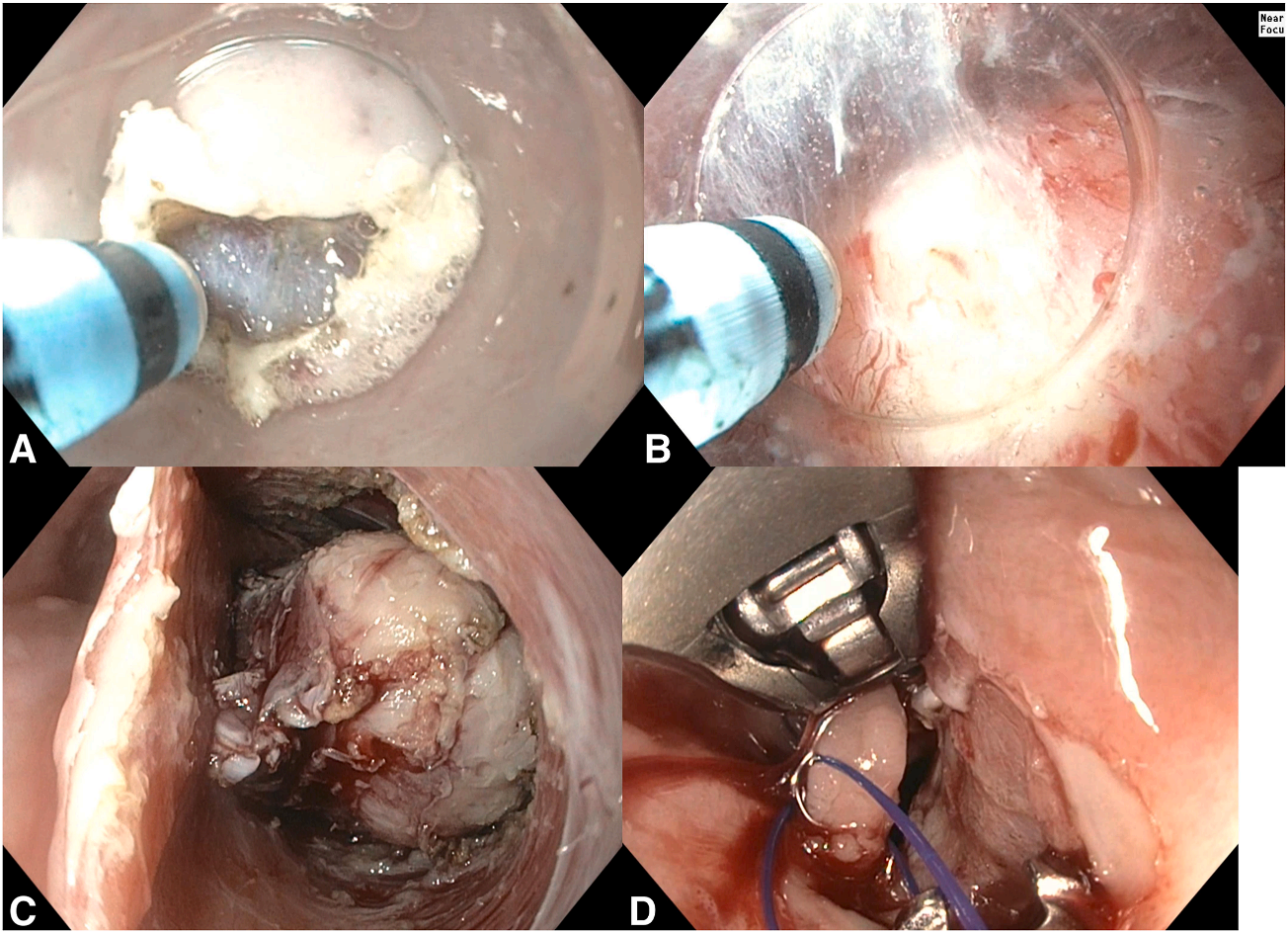
Submucosal tunneling endoscopic resection (*STER*) of a midesophageal schwannoma. A, Creation of the mucosal entry site. B, Submucosal tunnel formation with exposure of the submucosal plane. C, Intramural dissection demonstrating the tumor capsule within the muscularis propria. D, Endoscopic closure of the mucosal entry following incomplete endoscopic resection.

Submucosal tunneling endoscopic resection (STER) was initiated by the advanced gastroenterology team. The thoracic surgery team was present and on standby during the endoscopic portion as part of a coordinated multidisciplinary approach. A submucosal tunnel was created to expose the lesion (Figure 2, Video 1), and dissection was carried out to mobilize the tumor. Although significant progress was made in separating the mass from the overlying mucosal layer and developing the submucosal plane, substantial involvement of the muscularis propria and loss of a safe circumferential plane limited further endoscopic resection. Given the tumor size and depth, further dissection was believed to carry an increased risk of mucosal injury or incomplete resection, and the decision was made intraoperatively to proceed with robotic-assisted thoracoscopic resection.

**Figure 3:**
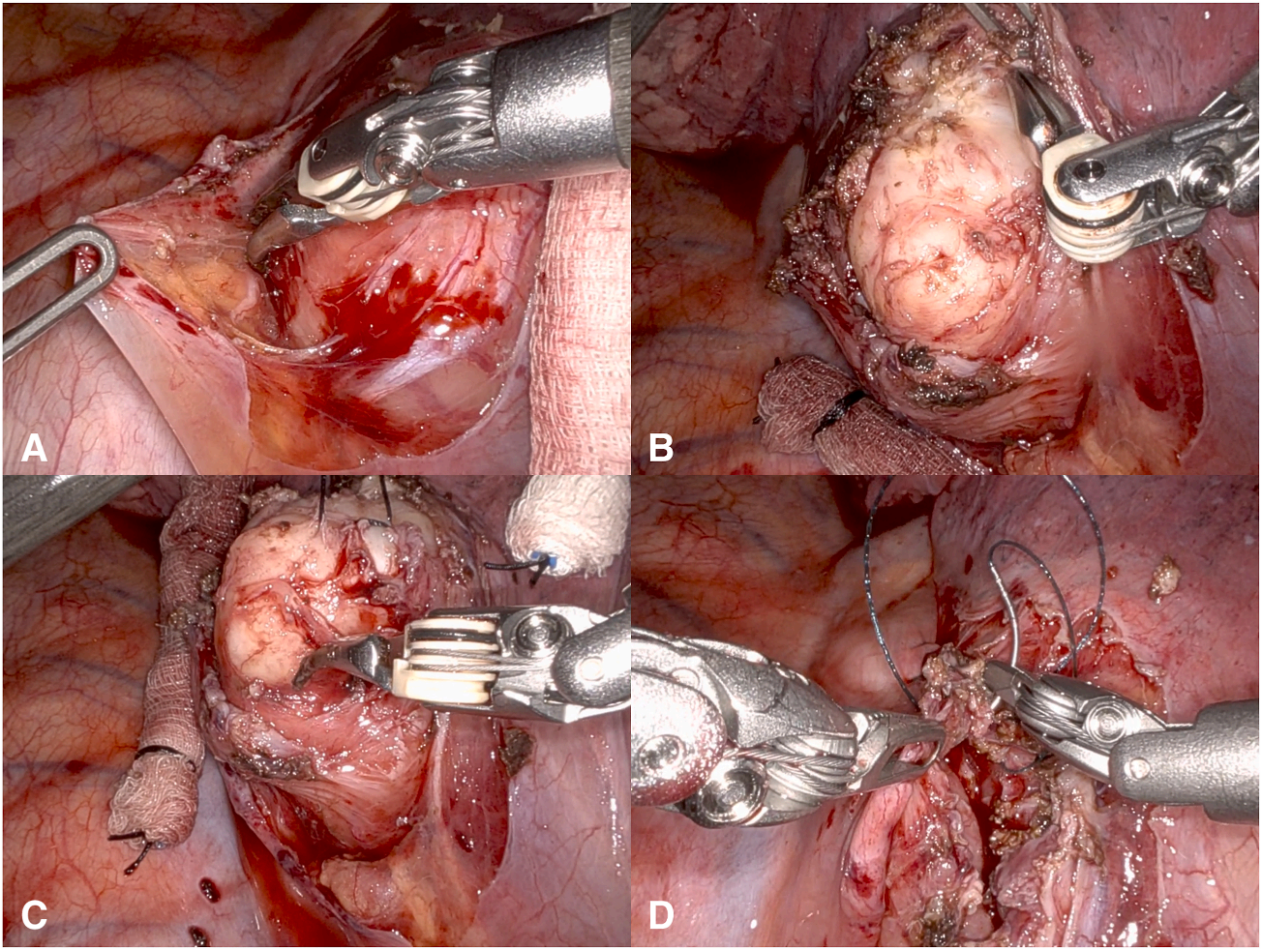
Robotic-assisted thoracoscopic resection of a midesophageal schwannoma. A, Opening of the mediastinal pleura overlying the esophagus. B, Dissection along the muscular layer to expose the intramural tumor. C, Mobilization and circumferential dissection of the submucosal schwannoma. D, Primary closure of the esophageal myotomy and pleural layer after complete tumor resection.

**Figure 4:**
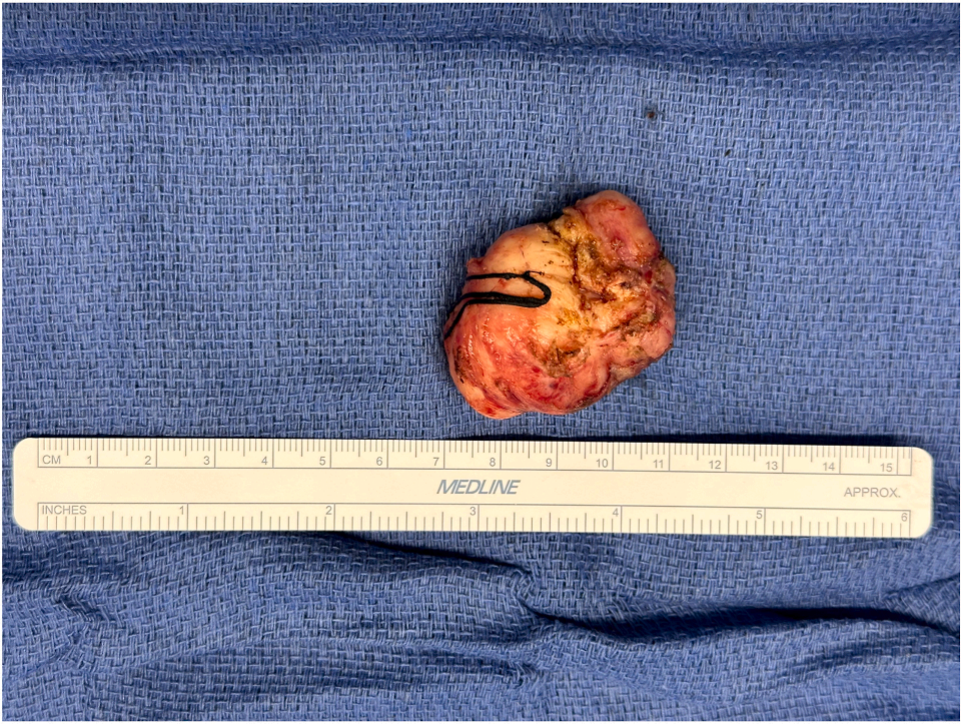
Gross specimen of the resected esophageal schwannoma. Encapsulated, tan-white, lobulated submucosal mass measuring approximately 4.7 × 2.9 cm, consistent with a benign esophageal schwannoma. A surgical marking stitch identifies the orientation of the specimen.

The endotracheal tube was exchanged for a double-lumen tube to facilitate single-lung ventilation, and the patient was repositioned in the left lateral decubitus position before robotic access was initiated. The right lung was reflected anteriorly, exposing the posterior mediastinum and the mass. The mediastinal pleura was incised (Figure 3, Video 2), and the esophageal muscle fibers were divided to expose the intramural tumor. The previous submucosal tunneling had already separated the tumor from the mucosa, creating a well-defined dissection plane. This facilitated robotic dissection by improving visualization, reducing the need for blunt dissection, and minimizing the risk of mucosal injury, a key concern in esophageal enucleation. Circumferential dissection was completed, and the specimen was removed Figure 4.

**Figure 5:**
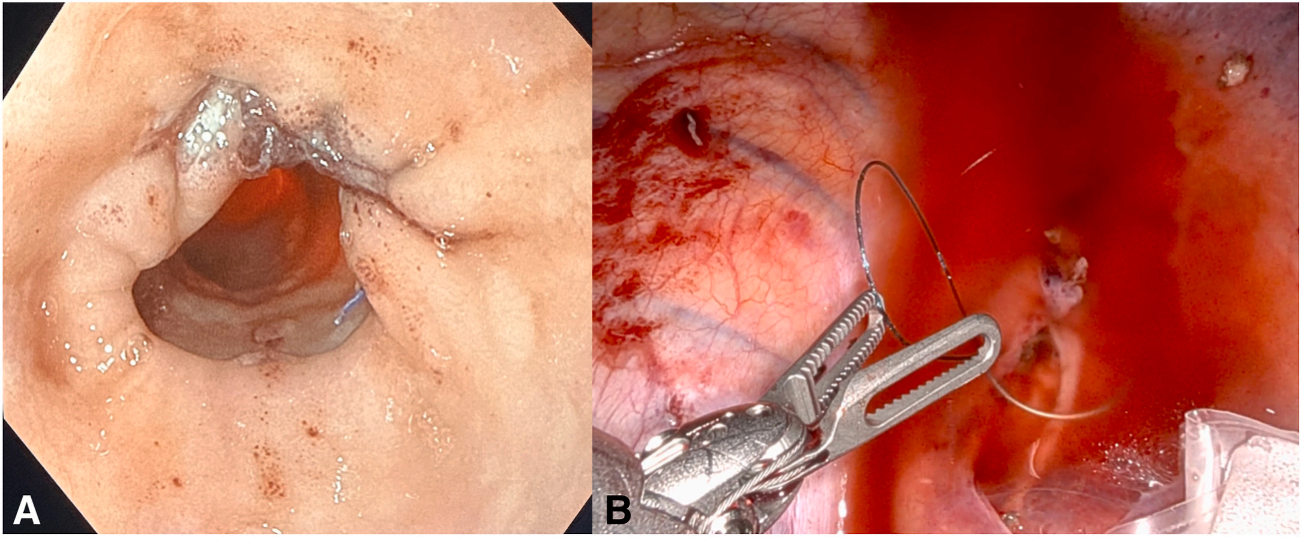
Intraoperative leak testing after hybrid endoscopic and robotic resection. A, Endoscopic air insufflation demonstrating an intact mucosal layer at the myotomy site with no evidence of intraluminal air leakage. B, Thoracoscopic view during simultaneous insufflation confirming a negative leak test, with no extraluminal bubbles or leakage observed.

The muscle layer was reapproximated with a running suture. Intraoperative endoscopy confirmed intact mucosa, a negative leak test (Figure 5), and adequate luminal patency. A chest tube was placed, and the lung was reinflated under direct visualization.

Total anesthesia time was approximately 5 hours. The endoscopic portion lasted 2 hours and 39 minutes, followed by a robotic portion lasting 1 hour and 42 minutes.

## Postoperative Course

The postoperative course was uncomplicated. An upper gastrointestinal contrast study demonstrated no leak. The patient advanced from a clear liquid diet to a regular diet without difficulty. The chest tube was removed on postoperative day 3, and she was discharged on postoperative day 4. At 2-week follow-up, she reported complete resolution of dysphagia.

## Discussion

Esophageal schwannomas are rare, accounting for less than 2% of all esophageal tumors.^4^ They most often arise in the upper or midesophagus and can present with dysphagia, weight loss, or be discovered incidentally.^2^ Histopathologic confirmation relies on S100 positivity and absence of other mesenchymal markers.^1^

Although robotic resection is a standard approach, the hybrid strategy may offer advantages in select cases. Submucosal tunneling during STER establishes a safe plane between the tumor and mucosa before surgical intervention, reducing the risk of mucosal injury during subsequent dissection.^5^ In this case, this facilitated efficient circumferential mobilization and likely contributed to the relatively short duration of the robotic portion.

Hybrid approaches may be particularly useful for candidates who are on the borderline for STER, including those with larger lesions or those with deeper muscular involvement in which complete endoscopic resection is uncertain. When a hybrid approach is anticipated, creation of a longer submucosal tunnel may help ensure adequate separation between the mucosal entry site and the operative field. In practice, a hybrid approach may be particularly valuable when endoscopic dissection successfully establishes a submucosal plane, but complete resection remains limited by tumor size or depth, allowing safe and efficient transition to minimally invasive surgical resection.

## Conclusions

This case demonstrates that a hybrid endoscopic and robotic-assisted approach can enable safe, minimally invasive resection of esophageal schwannomas while preserving mucosal integrity and achieving excellent clinical outcomes.

## Conflict of Interest Statement

The authors reported no conflicts of interest.

The *Journal* policy requires editors and reviewers to disclose conflicts of interest and to decline handling or reviewing manuscripts for which they may have a conflict of interest. The editors and reviewers of this article have no conflicts of interest.

## Supplementary Data

Video 1 Submucosal tunneling endoscopic resection (STER) demonstrating mucosal entry, submucosal tunnel creation, and dissection of the intramural esophageal mass from the overlying mucosal layer, establishing a submucosal plane while preserving mucosal integrity. Video available at: https://www.jtcvs.org/article/S2666-2507(26)00225-7/fulltext.

Video 2 Robotic-assisted thoracoscopic resection demonstrating esophageal myotomy, circumferential tumor mobilization, and completion of resection after previous submucosal tunneling, with preservation of the mucosal layer. Video available at: https://www.jtcvs.org/article/S2666-2507(26)00225-7/fulltext..
